# *Klebsiella variicola* improves the antioxidant ability of maize seedlings under saline-alkali stress

**DOI:** 10.7717/peerj.11963

**Published:** 2021-08-09

**Authors:** Lijuan Yang, Yufeng Wang, Kejun Yang

**Affiliations:** 1 College of Agronomy, Heilongjiang Bayi Agricultural University, Daqing, Heilongjiang, People’s Republic of China; 2 Key Laboratory of Crop Germplasm Improvement and Cultivation in Cold Regions of Education Department, Heilongjiang Bayi Agricultural University, Daqing, Heilongjiang, People’s Republic of China

**Keywords:** Active oxygen metabolism, Klebsiella variicola, Maize, Saline-alkali stress

## Abstract

**Background:**

Saline-alkali soil is mainly distributed in the northern and coastal areas of China. The Songnen Plain, located in the northeast of China, is a region with a relatively high concentration of saline-alkali soil and is also one of the more at-risk areas in the country. Every year, the increasing spread of saline-alkali soil areas has a serious impact on the growth of agricultural crops. The maize crop is sensitive to saline-alkali stress, which seriously affects its growth and development. Our previous study determined that *Klebsiella variicola* performs a variety of biological functions, as well as improves the rhizosphere microenvironment and promotes the growth and development of maize seedlings in saline-alkali soil environments. The present study further analyzed the mechanism that enables *K. variicola* to alleviate saline-alkali stress at the level of the antioxidant system.

**Methods:**

The accumulation of O_2_^−^ was observed directly via histochemical staining. The activities of several antioxidant enzymes were determined using the nitro blue tetrazolium and the guaiacol methods. The contents of non-enzymatic antioxidants were determined using the dithionitrobenzoic acid method.

**Results:**

The contents of the superoxide anion and hydrogen peroxide in leaves and roots of maize seedlings increased under saline-alkali stress conditions. The higher level of reactive oxygen species increased the degree of membrane lipid peroxidation. There were differences in the degree of oxidative damage and performance of the antioxidant defence system in maize seedlings under saline-alkali stress. Following the application of increasing concentrations of * K. variicola*, the activity of antioxidant enzymes increased by 21.22%–215.46%, and the content of non-enzymatic antioxidants increased as well, the ratios of ASA/DHA and GSH/GSSG in leaves increased by 4.97% and 1.87 times, respectively, and those in roots increased by 3.24% and 1.60 times, respectively. The accumulation of reactive oxygen species was reduced, and the content of H_2_O_2_ decreased by 26.07%–46.97%. The content of O_2_^−^ decreased by 20.18%–37.01%, which alleviated the oxidative damage to maize seedlings caused by saline-alkali stress.

**Conclusion:**

*K. variicola* reduced ROS-induced peroxidation to membrane lipids and effectively alleviated the damage caused by saline-alkali stress by increasing the activities of antioxidant enzymes in maize seedlings, thus enhancing their saline-alkali tolerance. A bacterial concentration of 1×10^8^ cfu/mL was optimal in each set of experiments.

## Introduction

The western part of the Songnen Plain has the largest amount of saline-alkali soil in China. Daqing City in Heilongjiang Province has the most severe saline-alkali soil problem in the Songnen Plain. The soil pH may exceed 10 and the Na^+^ content can surpass 0.9 g kg^−1^. These physicochemical conditions have a strong impact on crop growth. Saline-alkali stress adversely affects maize growth and development and may cause wilting at the seedling stage. Seedlings subjected to saline-alkali stress produce large quantities of reactive oxygen species (ROS) (Ahmad et al., 2010; Ahmad et al., 2019; Kohli et al., 2019) that gradually induce lipid peroxidation (Zhang, Liao & Yu, 2018; Ahmad et al., 2010; Ahmad et al., 2019; Kohli et al., 2019). This condition activates the antioxidant defence system in the plant (Ahmad et al., 2010; Ahmad et al., 2019; Kohli et al., 2019; Li, Tian & Gong, 2020). The antioxidant system consists of antioxidant enzymes such as SOD, POD, CAT, APX, GPX, and GR and non-enzymatic antioxidants such as ascorbic acid and glutathione. These can effectively eliminate excess ROS in plant cells and are, therefore, essential for maintaining intracellular redox balance and ensuring normal plant growth under adverse conditions (Yin, Wang & Li, 2013; Ahmad et al., 2010; Ahmad et al., 2019; Kohli et al., 2019).

Klebsiella variicola is a plant growth-promoting bacterium. It fixes nitrogen, dissolves phosphorus, produces IAA, and improves nutrition and stress tolerance in plants. Previous studies showed that plant growth-promoting bacteria regulate various pathways including the oxidation system and improve crop tolerance to saline-alkali stress (Jian, Liu & Li, 2017; Brotman, Landau & Álvaro, 2013; Ahmad et al., 2015; Parray, 2016). A study by Rawat, Singh & Shukla (2011) showed that under saline-alkali stress, antioxidant enzyme activity and other parameters increased to varying degrees in plants treated with *Trichoderma asperellum*. Enhancement of antioxidant enzyme activity accelerates the removal of superoxide anion and alleviates damage caused by hydrogen peroxide under abiotic stress. In this way, it significantly mitigates physiological damage to plants. Wang et al. (2021) showed that saline-alkali stress affected the non-enzymatic antioxidant levels and especially that of ascorbic acid (ASA) in crops. After 24 h saline-alkali stress, the ASA content was significantly reduced and its coding gene was markedly downregulated. Barriuso (2008) studied the ability of four plant growth-promoting bacteria to induce systemic resistance in *Arabidopsis thaliana*. All four bacterial strains enhanced plant resistance against the leaf pathogen *Pseudomonas syringae* DC3000 and improved plant tolerance to salt stress induced by 60 mM NaCl. The PDF1.2 gene related to the SA-independent pathway was upregulated in bacteria-treated plants. The defensive response of bacteria-treated plants was SA pathway-dependent. Banaei (2015) applied *Pseudomonas fluorescens* to canola roots and analysed the host proteome. The results showed that bacterial inoculation improved crop salt tolerance by increasing the levels of the proteins related to glycolysis, the tricarboxylic acid cycle, and amino acid metabolism. *Pseudomonas fluorescens* increased energy metabolism and cell division-related proteins in canola roots, thereby improving salt stress tolerance. Rice inoculated with *Bacillus amylolyticus* SN13 showed enhanced salt stress tolerance and growth. The bacteria upregulated ≥13 genes including *nadp-me2*, *erebp*, *sosi*, *badh*, and *serk1* associated with salt stress. They also promoted the betaine, sucrose, trehalose, and glutamine-utilizing pathways (Nautiyal, 2013).

Our previous study demonstrated that *Klebsiella variicola* performs various biological functions and improves the rhizosphere microenvironment and saline-alkali tolerance in maize seedlings (Yang & Yang, 2020). The present study explores the mechanisms by which the plant growth-promoting bacterium *Klebsiella variicola* regulates antioxidant enzymes and non-enzymatic antioxidants in maize. We also investigated the physiological mechanisms by which *K. variicola* enhances salt-alkali tolerance in maize seedlings. Hence, this study lays a theoretical foundation for maize cultivation and agricultural development in saline-alkali soil regions. Other objectives were to clarify the (1) changes in antioxidant enzyme activity and non-enzymatic antioxidant levels that occur in the leaves and roots of maize seedlings subjected to saline-alkali soil stress and (2) mitigation of ROS damage in plants inoculated with *K. variicola*.

## Material and Methods

### Preparation of bacteria

*Klebsiella variicola* (CGMCC1.15640) was acquired from the China General Microbiological Culture Collection Center (Beijing, China). *Klebsiella variicola* suspensions were collected as previously described in Yang & Yang (2020). The pellets were diluted using the following *K. variicola* treatments: K1, 1 × 10^2^ cfu/mL; K2, 1 × 10^4^ cfu/mL; K3, 1 × 10^6^ cfu/mL; and K4, 1 × 10^8^ CFU/mL.

### Experimental conditions

#### Pot experiment

Xianyu 335 maize seeds were purchased from Fuzun Agricultural Integrated Service Chain Co. Ltd. (Heilongjiang, China). Five seeds were sown per pot. Different concentrations of *Klebsiella variicola* suspensions (100 mL) were added to each pot and cultured in a light incubator for 15 d. The culture conditions were previously described in Yang & Yang (2020). Con1 (control) was unamended saline-alkali soil, K1 was 1 × 10^2^ cfu/mL, K2 was 1 × 10^4^ cfu/mL, K3 was 1 × 10^6^ cfu/mL, K4 was 1 × 10^8^ cfu/mL *Klebsiella variicola* added to saline-alkali soil, and Con2 (control) was unamended soil with normal salinity and alkalinity. The saline-alkali soil conditions were pH 9.2 and Na^+^ content = 0.906 g kg^−1^. The sampling time was 15 d after sowing.

#### Histochemical staining to observe H_2_O_2_ and O${}_{2}^{-}\cdot $ accumulation

Maize leaves were immersed in 2 mM nitro blue tetrazolium (NBT) and diaminobenzidine (DAB) solution under vacuum for 3 h to observe O${}_{2}^{-}\cdot $ and H_2_O_2_ accumulation, respectively, according to the method of Zhang, Lei & Hui (2011). Chlorophyll was removed by boiling the leaves in a 4:1 (v/v) mixture of 75% (v/v) ethanol and 5% (v/v) glycerol. The leaves were then observed and photographed.

#### Determination of H_2_O_2_ content

H_2_O_2_ was determined according to the method of Velikova, Yordanov & Edreva (2000). Each 0.5-g leaf sample was mixed with five mL of 0.1% (v/v) trichloroacetic acid and ground in an ice bath. Each extract (1–2 mL) was centrifuged at 6, 000 ×g for 15 min and mixed with 0.5 mL of 10 mM potassium phosphate buffer (pH 7.0) plus one mL of 1 mM KI. Absorbance was read at 390 nm.

#### Determination of O_2_^−^⋅ content

The O${}_{2}^{-}\cdot $ content was determined according to the method of Puyang, An & Han (2015). Each supernatant (one mL) was mixed with one mL of 1 mM hydroxylamine hydrochloride and incubated at 25 °C for 20 min. The mixture was then added to 0.2 mL of 170 mM *p*-aminobenzenesulfonic acid plus 0.2 mL of 70 mM *α*-naphthylamine and incubated at 25 °C for 20 min. An equal volume of ether was added and the mixture was shaken well and centrifuged at 1,500 × g for 5 min.

#### Determination of antioxidant enzyme activity

Superoxide dismutase (SOD) activity was determined according to the method of Giannopolitis & Ries (1977), with modifications. Each reaction mixture (three mL) contained 50 mM potassium phosphate buffer (pH 7.8), 75 µM NBT, 2 µM riboflavin, 13 M methionine, 0.1 mM EDTA-Na_2_, and 100 µL crude enzyme extract.

Peroxidase (POD) activity was measured according to the method of Hernández, Jiménez & Mullineaux (2000), with modifications. Each reaction mixture (three mL) contained 100 mM potassium phosphate buffer (pH 6.0), 10% (v/v) guaiacol, and 30% (v/v) H_2_O_2_. Grinding solution (one mL) was the control.

Glutathione peroxidase (GPX) activity was determined according to the method of Wang & Huan (2015), with modifications. Each reaction mixture (three mL) contained 50 mM potassium phosphate buffer (pH 7.0), 0.1 mM EDTA, 5 mM guaiacol, 15 mM H_2_O_2_, and 100 µL crude enzyme extract.

Catalase (CAT) activity was measured according to the method of Hui & Dang (2014), with modifications. Each control tube contained crude enzyme solution (0.2 mL), potassium phosphate buffer (1.5 mL), and distilled water (one mL). The tubes were placed in a boiling water bath for 1–2 min to denature the enzyme and then they were cooled. The tubes in the second set were prepared using the aforementioned reagents, and 0.3 mL of 0.1 M H_2_O_2_ was added to each tube.

Ascorbate peroxidase (APX) activity was determined according to the method of Jiang, Shen & Zhou (2013), with modifications. Each reaction mixture (three mL) consisted of 1.5 mL of 50 mM potassium phosphate buffer (pH 7.0), 0.1 mL of 15 mM ASA, 0.3 mL of 1 mM H_2_O_2_, and one mL of distilled water. Then 100 µL crude enzyme extract was added to each mixture to initiate the reaction.

Glutathione reductase (GR) activity was measured according to the method of Li, Chen & Zheng (2015), with modifications. Each reaction mixture (three mL) consisted of 50 mM potassium phosphate buffer (pH 7.8), 2 mM EDTA-Na_2_, 0.15 mM NADPH, 0.5 mM GSSG, and 100 µL crude enzyme extract. NADPH was added last to initiate the reaction. For the control, precooled enzyme extraction buffer was used instead of crude enzyme extract.

#### Determination of ascorbic acid content

Leaf tissue (0.5 g) was ground with 2.5 mL of 5% (w/v) sulfosalicylic acid and centrifuged at 5,000× g and 4 °C for 20 min. The ASA and GSH concentrations in the supernatants were measured according to the method of Fryer et al. (1998).

#### Determination of glutathione content

The glutathione (GSH and GSSG) content was measured according to the method of Nagalakshmi & Prasad (2001). Each reaction mixture consisted of 706 µL phosphate buffer, 20 µL NADPH (10 M), and 80 µL dithionitrobenzoic acid (12.5 M). After incubation at 25 °C for 10 min, 20 µL GR (50 U mL^−1^) was added to a total final volume of one mL. The solution was mixed and the absorbance was measured at 412 nm.

### Statistical analyses

One-way ANOVA was conducted in SPSS 21.0 (SPSS Inc., Chicago, IL, USA). Duncan’s test was used for multiple comparisons and analysis of the differences between treatment means. Significance was set to *P* < 0.05. All data presented in the tables are the averages of at least three replicates.

## Results

### Effect of *K. variicola* on non-enzymatic antioxidant content

Table 1 show that the ASA:DHA and GSH:GSSG ratios in the leaves and roots of maize plants grown under saline-alkali stress conditions (Con1) were significantly lower than those in the same organs of plants grown under non-saline-alkali conditions (Con2). Moreover, the ASA:DHA and GSH:GSSG ratios were higher in the leaves than the roots of Con1. Relative to Con1, foliar AsA:DHA and GSH:GSSG had decreased by 88.44% and 60.19%, respectively. The total ascorbic acid and total glutathione levels were significantly higher in the leaves and roots of Con1 than they were in the same organs of Con2. For the leaves and roots of the plants subjected to saline-alkali stress, the ASA:DHA ratios were higher than the GSH:GSSG ratios.

**Table 1:** Effects of *Klebsiella variicola* on non-enzymatic antioxidants in leaves and roots of maize grown on saline-alkaline soil. Con1 is saline-alkali soil unadded control group, K1 is 1 × 10^2^, K2 is 1 × 10^4^, K3 is 1 × 10^6^, K4 is 1 × 10^8^ cfu/mL concentration of Klebsiella variicola added saline-alkali soil, Con2 is nonsaline-alkali soil unadded control group.

**Sampling part**	**Treatment**	**ASA + DHA** **(µmol g** ^−1^ **FW)**	**ASA / DHA**	**GSSG + GSH** **(µmol g** ^−1^ **FW)**	**GSH / GSSG**
Leaf	Con1	3.52 ± 0.111d	0.34 ± 0.019d	215.18 ± 4.527e	2.93 ± 0.463d
	Con2	2.92 ± 0.145e	2.94 ± 0.112a	183.70 ± 3.889f	7.36 ± 0.311a
	K1	4.32 ± 0.268c	0.38 ± 0.037d	254.06 ± 0.575d	3.17 ± 0.164d
	K2	4.95 ± 0.172b	0.95 ± 0.067c	269.78 ± 5.348c	3.83 ± 0.165c
	K3	5.42 ± 0.204a	1.69 ± 0.296b	291.91 ± 4.841b	4.33 ± 0.366c
	K4	5.32 ± 0.162a	1.93 ± 0.187b	306.03 ± 2.006a	5.47 ± 0.406b
Root	Con1	2.71 ± 0.166d	2.68 ± 0.249d	225.33 ± 9.271e	4.22 ± 0.302d
	Con2	2.33 ± 0.051e	9.49 ± 0.271a	189.88 ± 3.152f	9.07 ± 0.651a
	K1	3.09 ± 0.069c	2.99 ± 0.186d	259.73 ± 2.354d	5.77 ± 0.029c
	K2	4.19 ± 0.284b	5.19 ± 0.875c	281.04 ± 3.765c	5.84 ± 0.111c
	K3	4.98 ± 0.136a	6.83 ± 0.581b	300.55 ± 6.711b	6.07 ± 0.892bc
	K4	5.08 ± 0.162a	8.67 ± 0.201a	312.73 ± 3.288a	6.76 ± 0.066b

The leaf and root total ascorbic acid and total glutathione levels and the ASA:DHA and GSH:GSSG had significantly increased with applied *K. variicola* concentration. There were no significant differences between K3 and K4 in terms of foliar total ascorbic acid or ASA:DHA ratio. Relative to Con1 the total ascorbic acid and total glutathione levels in the leaves increased by 53.98% and 42.22%, respectively. In the roots, the total ascorbic acid and total glutathione content increased by 83.76% and 38.79%, respectively. The foliar ASA:DHA and GSH:GSSG ratios increased by 397.06% and 86.69%, respectively, whilst the root ASA:DHA and GSH:GSSG ratios increased by 223.51% and 60.19%, respectively. Hence, *K. variicola* application positively influenced the maize seedlings by alleviating saline-alkali stress, increasing the foliar non-enzymatic antioxidant content and ratios, mitigating the damage caused by the ASA-GSH cycle, improving the intracellular redox potential, enhancing the cellular antioxidant capacity, and maintaining a balanced state of active oxygen metabolism.

### Effects of *K. variicola* on ROS accumulation in maize seedlings under saline-alkali stress

**Figure 1:**
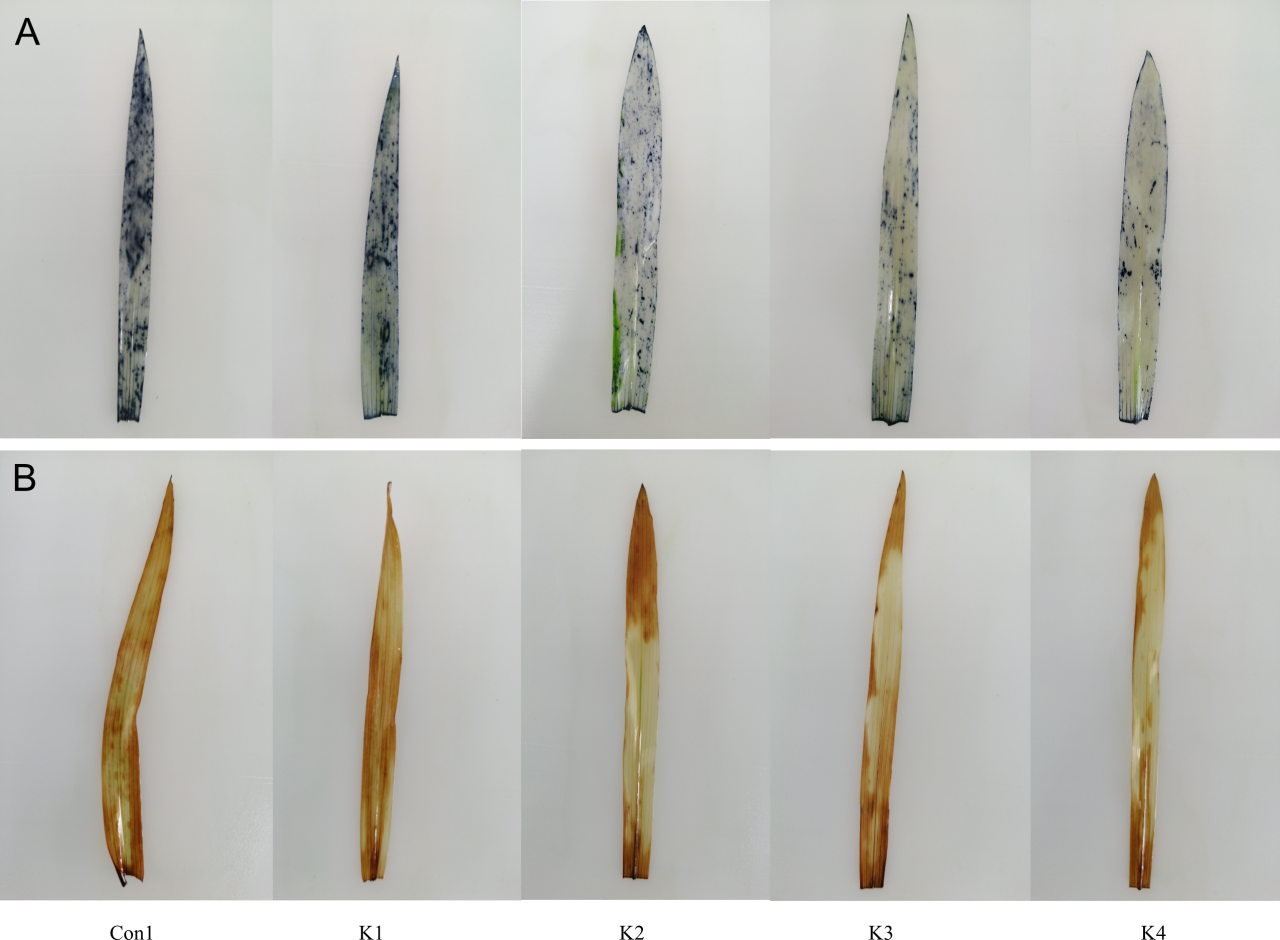
Influence of *K. variicola.* on O ${}_{2}^{-\cdot }$ and H_2_O_2_ levels in the leaves of maize plants under saline–alkaline. Con1 is the control group; K1, K2, K3, and K4 correspond to a concentration of 1 × 10^2^, 1 × 10^4^, 1 ×10^6^, and 1 × 10^8^ cfu/mL of *Klebsiella variicola*, respectively. (A) Staining with NBT, (B) staining with DAB.

**Figure 2:**
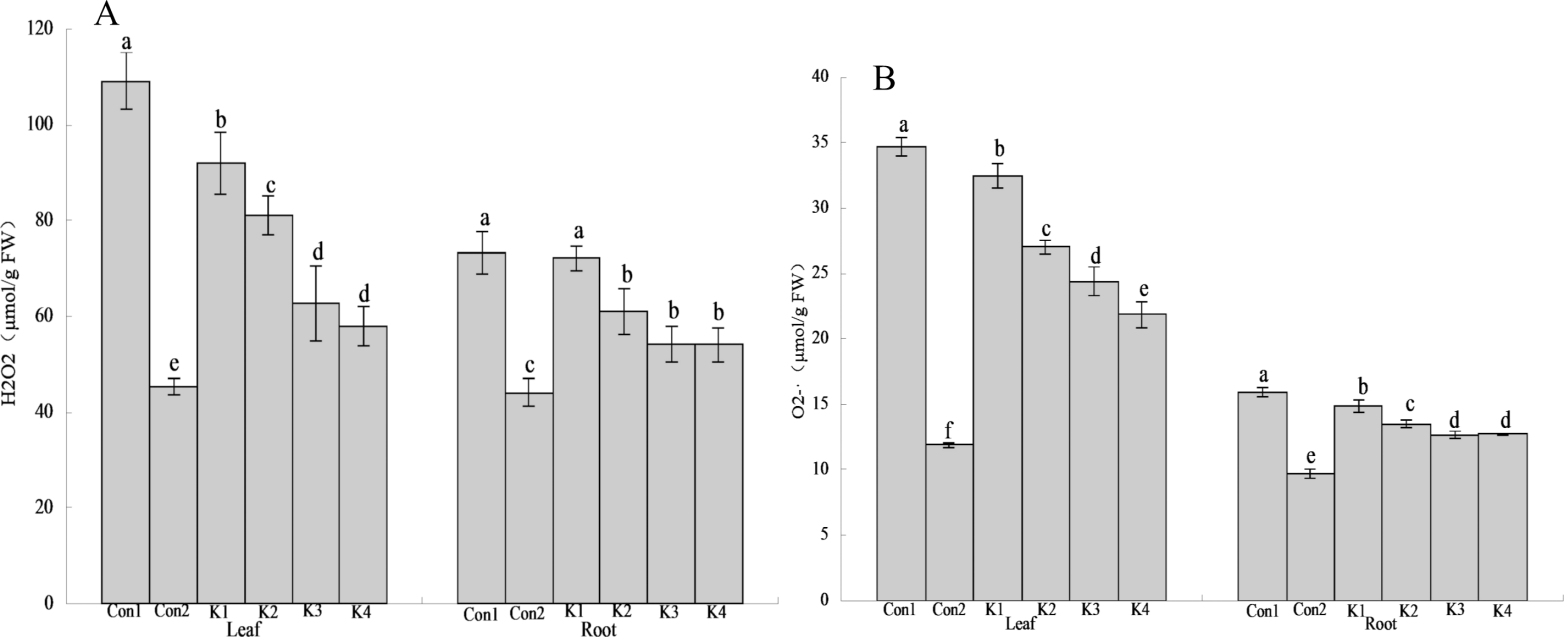
Influence of *K. variicola.* on H_2_O_2_ and O ${}_{2}^{-\cdot }$ levels in the leaves and roots of maize plants under saline–alkaline. Con1 is saline-alkali soil unadded control group, K1 is 1 × 10^2^, K2 is 1 × 10^4^, K3 is 1 ×10^6^, K4 is 1 × 10^8^cfu/mL concentration of *Klebsiella variicola* added saline-alkali soil, Con2 is nonsaline-alkali soil unadded control group. (A) H2O2 content, (B) O2 content.

Staining with NBT and DAB revealed O${}_{2}^{-}$ and H_2_O_2_ accumulation, respectively. The colour intensity of the leaves of maize plants subjected to saline-alkali stress and treated with *K. variicola* decreased with increasing bacterial concentration and was lower than that of the control (Con1) (Figs. 1 and 2). Thus, *K. variicola* application gradually lowers O${}_{2}^{-}\cdot $ and H_2_O_2_ content in maize leaves and prevents the excessive accumulation of these ROS induced by saline-alkali stress.

**Figure 3:**
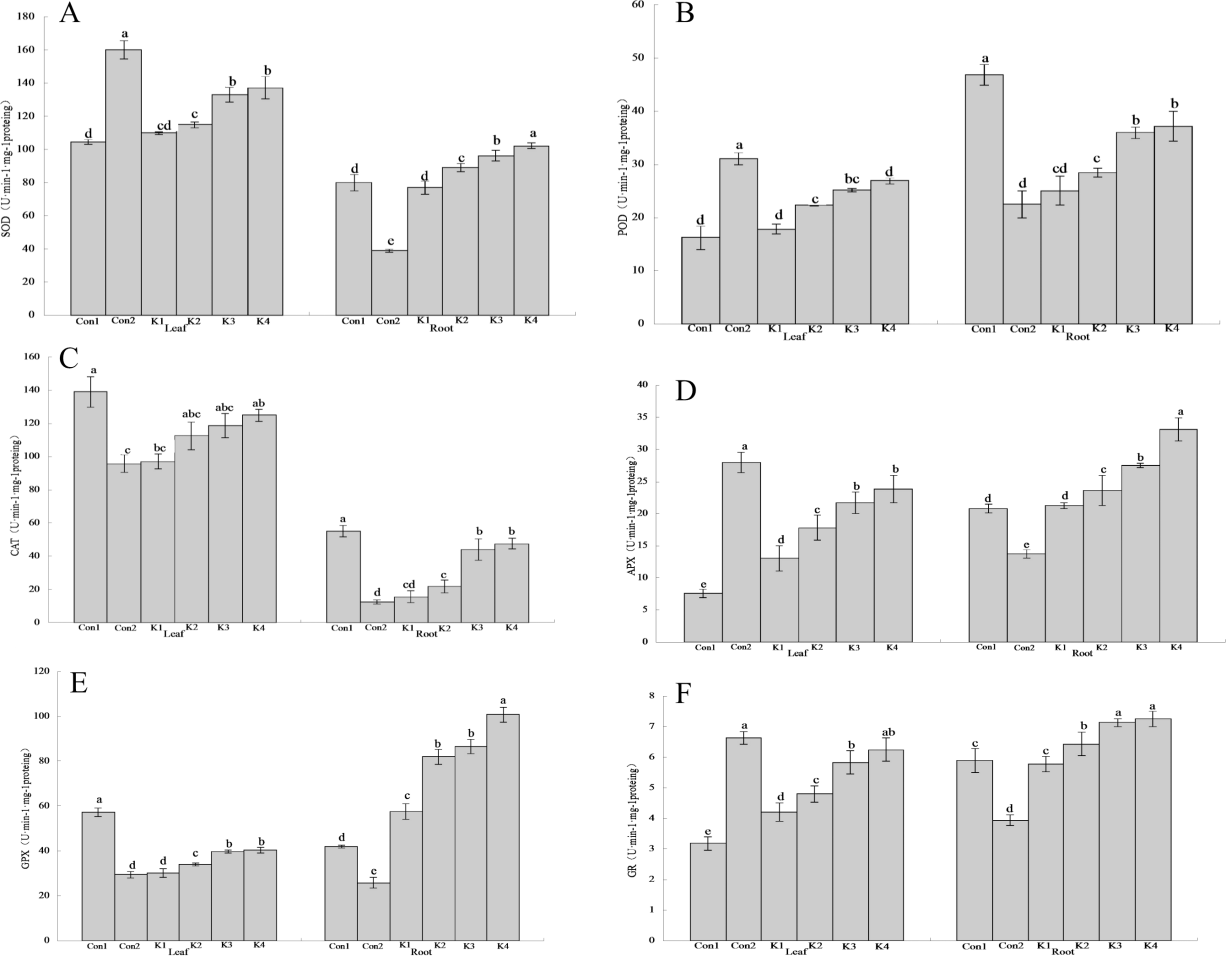
Influence of *K. variicola* on the antioxidant enzyme activities in the leaves and roots of maize seedlings in saline-alkaline soil. Con1 is saline-alkali soil unadded control group, K1 is 1 × 10^2^, K2 is 1 × 10^4^, K3 is 1 ×10^6^, K4 is 1 × 10^8^cfu/mL concentration of *Klebsiella variicola* added saline-alkali soil, Con2 is nonsaline-alkali soil unadded control group. (A) SOD content, (B) POD content, (C) CAT content, (D) APX content, (E) GPX content, (F) GR content.

The H_2_O_2_ content in the leaves and roots of maize under saline-alkali stress conditions (Con1) was significantly higher than it was in the same organs of the plants under normal conditions (Con2). The H_2_O_2_ levels in the leaves and roots were 140.90% and 66.44% higher than normal conditions (Con2), respectively, in Con1. Treatment with various concentrations of *K. variicola* lowered H_2_O_2_ accumulation in the leaves and roots and alleviated the cell membrane peroxidation induced by saline-alkali stress. The K3 and K4 treatments were significantly more efficacious than the other treatments. Compared with the control, H_2_O_2_ accumulation in the leaves under the K3 and K4 treatments had decreased by 42.58% and 46.97%, respectively, whereas H_2_O_2_ accumulation in the roots under the K3 and K4 treatments had decreased by 26.07% and 26.21%, respectively. Therefore, *K. variicola* application in saline-alkali soils can fortify maize crop resistance to saline-alkali stress. H_2_O_2_ accumulation decreased to a greater extent in the leaves than the roots (Fig. 3). Hence, the bacteria had higher protective efficacy on the leaves than the roots.

The O${}_{2}^{-}\cdot $ content was higher in the leaves than the roots. It was also higher under saline-alkali stress conditions than it was under normal conditions (Fig. 3). Treatment with *K. variicola* alleviated O${}_{2}^{-}\cdot $ accumulation in the leaves and roots, but to a greater extent in the former than the latter. The foliar O${}_{2}^{-}\cdot $ content under the K4 treatment was 37.01% lower than that for the control. No significant difference was detected between the K3 and K4 treatments in terms of their root O${}_{2}^{-}$ levels. They were 20.30% and 20.18% lower, respectively, than that of the control.

### Effects of *K. variicola* on antioxidant enzyme activity in maize seedlings under saline-alkali stress

The SOD activity in the leaves of maize grown under saline-alkali stress conditions (Con1) was 53.15% lower than that in the leaves of maize grown under normal conditions (Con2) (Fig. 3A). By contrast, the SOD activity in the roots of Con1 was 105.10% higher than that in the roots of Con2. After *K. variicola* application, the SOD activity had increased in the leaves and roots, and the overall SOD activity increased with bacterial concentration. At the lower bacterial concentrations, the enzyme activity did not significantly differ from that of Con1. At the higher bacterial concentrations, however, the enzyme activity was significantly higher than that of Con1. The K3 and K4 treatments were significantly more efficacious on the leaves than the other treatments. The foliar SOD activity levels were 27.39% and 31.46% higher for the K3 and K4 treatments, respectively, than that of the control. The root SOD activity level was 27.77% higher for the K4 treatment than the control.

The foliar POD activity for Con1 was 90.95% lower than that of Con2 (Fig. 3B). By contrast, the root POD activity for Con1 was 108.40% higher than that for Con2. After *K. variicola* application, the relative POD activity had increased with bacterial concentration in both the leaves and the root. There was no significant difference between K1 and Con1 in terms of leaf POD activity. However, the remaining treatments resulted in significantly higher POD activity than that of Con1. The K4 treatment was significantly more efficacious on the leaves than the other treatments. Foliar SOD activity was 65.72% higher in the K4 treatment than the control. The K3 and K4 treatments markedly increased root POD activity (30.25% and 26.15%, respectively) compared with the control (Con1). Nevertheless, there was no significant difference between K3 and K4 in terms of root POD activity.

The CAT activity levels in the leaves and roots of Con1 were significantly (*P* < 0.05) higher than those in the same organs of Con2 (45.08% and 344.43% higher, respectively; Fig. 3C). In general, the CAT activity was higher in the leaves than the roots. The overall leaf and root CAT activity increased with *K. variicola* concentration. K4 was significantly more efficacious on the leaves than the other treatments. The foliar CAT activity was 10.11% lower for K4 than Con1. The root CAT activity levels in the K3 and K4 treatments were 19.99% and 13.70% lower, respectively, than that of Con1.

Foliar APX activity was significantly lower (72.90%) in Con1 than Con2. Conversely, root APX activity was significantly higher (51.13%) in Con1 than Con2 (Fig. 3D). Leaf and root APX activity increased with *K. variicola* concentration. Root APX activity did not significantly differ between K1 and Con1. However, all other treatments significantly increased root APX activity relative to Con1. The K3 and K4 significantly increased foliar APX activity compared with the other treatments (186.13% and 215.46%, respectively, relative to the control). K4 increased root activity by 59.29% compared with the control.

The leaf and root GPX activity levels in Con1 were significantly higher than those in Con2 (94.76% and 62.63%, respectively; Fig. 3E). Both the leaf and root GPX activity levels increased with *K. variicola* concentration. Nevertheless, the increase was greater in the roots than the leaves, and the maximum relative gain in root GPX activity was 139.96%. The lower bacterial concentrations did not significantly alter GPX activity compared with Con1. However, all other treatments were significantly more efficacious than Con1. Root GPX activity was 139.96% higher for K4 than the control.

Foliar GR activity was significantly lower for Con1 than Con2 (51.96%). Conversely, root GR activity was significantly higher for Con1 than Con2 (49.49%; Fig. 3F). The root and leaf GR activity increased with *K. variicola* concentration. There was no significant difference in GR activity between the low bacterial concentration (K1 treatment) and Con1. The lower bacterial concentrations did not significantly alter GR activity compared with Con1. The K4 treatment was significantly more efficacious than the others and increased foliar GR activity by 95.92% compared with the control. The K3 and K4 treatments increased GR activity 21.22% and 23.26%, respectively, relative to the control.

## Discussion

In plants, saline-alkali stress leads to ROS accumulation and disrupts normal dynamic ROS balance (Liao, 2019). Thence, the activity levels of enzymatic and non-enzymatic antioxidants increase in the attempt to attenuate the toxic effects of ROS (Ahmad et al., 2016; Kaur, 2018; Ahanger, 2020; Kaya et al., 2020).

Here, we found that the ratios of non-enzymatic antioxidants were markedly lower in the leaves than the roots. The APX and GR activity levels were higher in the roots than the leaves. Elevated root GR activity maintains normal ASA-GSH cycle performance and enables rapid transformation of GSSG and DHA into GSH and ASA, respectively. Similar results were reported for previous studies. Gu (2018) showed that non-enzymatic antioxidant activity significantly differed among various maize organs. Total ascorbic acid was higher in the leaf than the root whereas total glutathione was greater in the root than the leaf. Wang et al. (2021) demonstrated that the antioxidant enzyme levels significantly increased in wheat seedlings subjected to salt stress, but not equally in all organs and tissues. Moreover, the non-enzymatic antioxidant content also changed not equally in all organs and tissues in response to salt stress.

Previous studies showed that plants raised under saline-alkali stress conditions presented with increases in antioxidant enzyme activity (Pravej et al., 2019; Kaya et al., 2020). Another study indicated that the activity levels of certain antioxidant enzymes did not increase in response to saline-alkali stress (Parvaiz et al., 2019). Each antioxidant enzyme makes a different contribution in ROS scavenging. However, not all these enzymes must necessarily increase in activity to be efficacious (Ahmad et al., 2016; Kaur, 2018; Ahanger, 2020; Kaya et al., 2020). Our experimental results revealed that the trends in antioxidant enzyme activity in response to saline-alkali stress were generally similar for both roots and leaves. Nevertheless, there were a few differences. For maize under unamended saline-alkali stress conditions (Con1), the changes in foliar antioxidant enzyme activity were the opposite of those in the roots. ROS production induced by saline-alkali stress exceeded the capacity of the antioxidant system in the leaves and resulted in serious oxidative damage (Ahanger et al., 2018). The root ROS content also increased in response to saline-alkali stress, but to a lesser extent than that in the leaf. The root system simultaneously secreted certain compounds that conferred a measure of protection against oxidative damage. The observed increases in the activity of antioxidant enzymes such as CAT in the leaves and roots may be the result of ROS accumulation.

Here, maize seedling leaf and root SOD, APX, GPX, and GR activity increased with *K. variicola* concentration. The total ascorbic acid and total glutathione content as well as the GSH:GSSG and ASA:DHA ratios significantly increased in the leaves and roots after *K. variicola* treatment. The bacterial treatments also lowered the ROS content in the leaves and roots, but to a greater extent in the former than the latter. These findings indicated that in maize seedlings under saline-alkali stress, *K. variicola* could effectively improve the antioxidant capacity of the leaves and roots, increase their reduced ascorbic acid and glutathione content, and mitigate cell membrane damage caused by ROS. *Klebsiella variicola* more effectively attenuated the harmful effects of ROS on the leaves than the roots because it colonizes maize roots and the rhizosphere, regulates antioxidant enzyme gene transcription with its own metabolites, and upregulates SOD, POD, and other enzymes. Certain transcription factors play important roles in plant stress resistance (Yamaguchi & Shinozaki, 2014). Analysis of the *RD* promoter showed that a DRE-responsive element commonly occurs in the promoters of the genes responding to salt stress and regulates gene induction and expression under stress conditions. The DREB (re-binding protein) transcription factor induces target genes by binding with DRE *cis*-acting elements (Zhang, 2010). DREB1a induced antioxidant gene expression in Arabidopsis under saline-alkali stress (Pellegrineschi, 2004). Plant growth-promoting bacteria control the activity of antioxidant enzymes by regulating the promoters of antioxidant response genes.

Treating maize seedlings with increasing *K. variicola* concentrations was relatively more effective at alleviating saline-alkali stress. High bacterial concentrations were more efficacious than low bacterial concentrations. The optimal treatment was K4 which corresponded to 1 × 10^8^ cfu/mL bacterial suspension. *Klebsiella variicola* has potent growth-promoting efficacy. Concentrated bacterial suspensions are relatively more conducive to maize seedling production and have comparatively higher antioxidant capacity. Yang et al. (2016) showed that *Klebsiella variicola* synthesizes auxin (IAA) which ameliorates saline-alkali stress in plants. However, there were no significant difference between the K3 (1 × 10^6^ cfu/mL) and K4 (1 × 10^8^ cfu/mL) treatments in terms of foliar SOD, POD, or CAT activity. Hence, adequate levels of certain antioxidant enzymes were attained with 1 × 10^6^ cfu/mL *K. variicola*. Antioxidant enzyme activity was not significantly improved with further increases in bacterial suspension concentration. Yang et al. (2016) showed that when plants are challenged by their external environment, they exhibit antioxidant enzyme response thresholds. After these levels are reached, there is no significant change in antioxidant enzyme gene expression or protein activity.

## Conclusion

The present study demonstrated that the superoxide anion (O ${}_{2}^{-}$) and hydrogen peroxide (H_2_O_2_) content increased in the leaves and roots of maize seedlings under saline-alkali stress. Relative to the control, the H_2_O_2_ increased by 140.90% in the leaves and 66.44% in the roots of the saline-alkali-stressed maize seedlings. *Klebsiella variicola* increased antioxidant enzyme activity in maize seedlings under saline-alkali stress conditions. In response to the bacterial treatment, APX increased in the leaves by 215.46% whilst GPX increased in the roots by 139.96% to mitigate ROS-induced membrane lipid peroxidation and alleviate the damage caused by saline-alkali stress. In this way, the bacterial treatment improved saline-alkali tolerance in the maize seedlings. The optimal *K. variicola* concentration was determined to be 1 × 10^8^ cfu/mL. This study lays a theoretical foundation for the effective management of maize cultivation under adverse environmental conditions. The present study conducted pot experiments to test the efficacy of *Klebsiella variicola* at protecting maize seedlings against saline-alkali stress. However, future research should be conducted at the field level, and changes in antioxidant enzyme activity should be measured at all stages of maize development. Moreover, molecular-level experiments should be performed to determine maize antioxidant enzyme gene expression after *Klebsiella variicola* application.

##  Supplemental Information

10.7717/peerj.11963/supp-1 Supplemental Information 1 Raw measurements

## Additional Information and Declarations

### Data Availability

The following information was supplied regarding data availability: Raw measurements are available as a Supplementary File.
